# The Frequencies distribution of CYP3A5 rs776746 and ABCB1 rs1045642 polymorphisms in the west Algerian population and relationships with pharmacogenetics

**DOI:** 10.4314/ahs.v24i1.36

**Published:** 2024-03

**Authors:** Amina Ammour, Meriem Aberkane, Abdallah Boudjema, Wefa Boughrara, Sounnia Mediene Benchekor

**Affiliations:** 1 Laboratoire de Génétique Moléculaire et Cellulaire (LGMC), Université des Sciences et de la Technologie d'Oran Mohamed BOUDIAF (USTO-MB), BP 1505, El M'naouer, 31000 Oran, Algeria; 2 Department of Pharmacy, Faculty of Medicine, University of Oran 1 Ahmed Ben Bella, 31000 Oran, Algeria; 3 Service de cytogénétique et de biologie moléculaire, Etablissement hospitalo-universitaire d'Oran (EHUO); 4 École Supérieure en Sciences Biologiques d'Oran (ESSBO), BP 1042, Saim Mohamed 31003, Oran, Algeria; 5 Department of Biotechnology, Faculty of Natural and Life Sciences, University of Oran 1 Ahmed Ben Bella, 31000 Oran, Algeria

**Keywords:** CYP3A5, ABCB1, genetic polymorphism, pharmacogenetics, west Algeria

## Abstract

**Introduction:**

Pharmacogenetic markers, such as the ATP Binding Cassette (ABCB1) and cytochrome P450 (CYP) 3A5 enzymes, play a crucial role in personalized medicine by influencing drug efficacy and toxicity based on individuals' or populations' genetic variations.

This study aims to investigate the genetic polymorphisms of CYP3A5 (rs776746) and ABCB1 (rs1045642) in the West Algerian population and compare the genotypes and allelic distributions with those of various ethnic groups.

**Methods:**

The study involved 472 unrelated healthy subjects from the Western Algerian population. DNA genotyping was performed using TaqMan allelic discrimination assay. The variants in our population were compared to those in other ethnic groups available in the 1000 Genomes Project. Genotype and allele frequencies were calculated using the chi-square test and the Hardy–Weinberg equilibrium (HWE)

**Results:**

The minor allele frequencies were found to be 0.21 for CYP3A5 6986A and 0.34 for ABCB1 3435T. These frequencies were similar to those observed in North African populations, while notable differences were observed in comparison to certain Caucasian and African populations.

**Conclusion:**

The difference in the allelic and genotypic distribution of these polymorphisms emphasize the need for dose adjustments in drugs metabolized by CYP3A5 and transported by ABCB1 to optimize treatments outcomes.

## Introduction

Pharmacogenetics is a field of study of immense importance in comprehending how individuals respond to medications and maximizing treatment effectiveness 1. The presence of genetic variation, especially within drug-metabolizing enzymes, drug targets, and drug transporters, significantly influences the interindividual and interethnic variations observed in drug responses. Among the various types of genetic variations, single nucleotide polymorphisms (SNPs) stand out as the most prevalent 2. These SNPs have the potential to modify enzyme activity and thereby affecting the drug metabolism process 2.

The P-glycoprotein (P-gp) is a membrane of the ATP Binding Cassette (ABC) transporter family responsible for the efflux transport of a multitude of therapeutic molecules. This transporter, present on the surface of multiple cells in the body, utilizes ATP hydrolysis to allow the passage from the intracellular to the extracellular space 3. Several studies have demonstrated an association between ABCB1 genetic variation and the enzyme activity 4, 5. The most common polymorphisms are ABCB1 3435C>T (rs1045642), a substitution from cytosine (C) to thymine (T) that does not lead to any amino acid change but is associated with a decrease P-gp expression and protein stability 6. The TT genotype was associated with lower expression of the transporter than the CT and CC genotypes 6.

Cytochrome P450 3A5 (CYP3A5) is a significant pharmacogenetics marker 7. This enzyme belongs to the CYP3A family involved in the metabolism of more than 50% of currently prescribed drugs and the biodegradation of toxins 8. Genetic polymorphisms affecting CYP3A5 enzyme contribute to interindividual and interethnic variability in substrate metabolism 8.

Among the 11 identified SNPs in CYP3A5 gene, the 6986A>G (rs776746) has been extensively studied. It consists of the replacement of an adenine (A) by the guanine base (G) at position 6986 in intron 3 9. This SNP creates a splice acceptor site in the pre-mRNA, and results in the incorporation of 131bp of sequence from intron 3 into the mRNA 10. This results in the generation of an aberrantly spliced mRNA. This frameshift leads to premature termination at amino acid residue 102 with loss of enzyme activity 10.

Homozygosity for the CYP3A5 6986G allele produce a nonfunctional truncated protein, without any enzymatic activity 11.

The ABCB1 c.3435C>T and CYP3A5 c.6986A>G polymorphisms contribute to interindividual and interethnic variability in enzymes, leading to differences in drug response 8, 11. Investigating the distribution of these polymorphisms in the general population will facilitates the development of more effective drugs and appropriate dosing regiments based on an individual's genetic profile.

## Materials and methods

### Study population

Our study includes 472 healthy subjects aged between 18 and 70 years old, originated from the west of Algeria. Informed written consent was obtained from all participants prior to their inclusion. Furthermore, all the subjects are unrelated and had no known history of diseases. Blood samples were collected from donors at the Blood Transfusion Center (CTS) in Oran between the years 2019 and 2020, while additional samples were obtained from the DNA biobank established at the Laboratory of Genetics and Molecular and Cellular Biology at the University of Sciences and Technology (USTO) of Oran.

### DNA extraction and genotyping

A total of 5 mL of peripheral blood was collected from each participant using EDTA tubes and subsequently stored at -20°C. Genomic DNA was extracted using the salting out method 12.

The quality and quantity of DNA were assessed using the Cary 60 UV-Vis Spectrophotometer (Agilent, USA).

Molecular genotyping of SNPs was performed using TaqMan SNP genotyping assay (Applied Biosystems, USA) (Table 1) on qTOWER^3^ (Analytik Jena, Germany). In total 20 µl PCR reaction contained 1X TaqMan genotyping master mix (Applied Biosystems, USA), 1X SNP genotyping assay, and 20 ng DNA. PCR was performed at 95°C for 30 sec predenaturation 95°C for 10 sec for denaturation, followed by 50 cycles of denaturation at 95°C for 15 sec each, and annealing/extension at 60°C for 90 sec. The PCR products were measured at 60°C for 30 sec. Appropriate negative and positive controls were used.

**Table 1:** Details of the SNP used in the study

SNP	db SNP ID	Location	Assay sequence	SNP Type
ABCB1 c.3435C>T	rs1045642	Chr.7: 87509329	TGTTGGCCTCCTTTGCTGCCCTCAC**[A/G]** ATCTCTTCCTGTGACACCACCCGGC	Silent Mutation, Intragenic
CYP3A5 c.6986A>G	rs776746	Chr.7: 99672916	ATGTGGTCCAAACAGGGAAGAGATA**[T/C]** TGAAAGACAAAAGAGCTCTTTAAAG	UTR 5, Intron,

Table 1 presents the candidate SNPs analyzed. The table provides information such as the SNP ID, location on the chromosome, assay sequence, and the type of SNP.

### Statistical analyses

The Hardy Weinberg equilibrium test (HWE) was performed for each polymorphism. The chi-square test allowed the comparison of genotype and allelic frequencies with other populations. Statistical analysis was performed using IBM SPSS Statistics 21, P < 0.05 was considered statistically significant. Considering the number of tests performed, it was stated that the Bonferroni correction threshold for significance of association is 0.025.

Allele and genotype frequencies for each of the five continental populations were obtained from the 1000 Genomes Project (www.ensembl.org).

## Results

### Genotype and allelic frequencies

The genotype and allelic frequencies of CYP3A5c.6986A>G are reported in (table2). For CYP3A5c.6986A>G the homozygote genotype frequency for 6986GG was 62.5%, 5.9% for the wild type 6986AA and 31.6% for heterozygote type 6986GA. The distribution of allele frequencies was 78.3% concerning 6986G and 21.7% for 6986A.

**Table 2:** Genotypes and alleles distribution of CYP3A5 c.6986A>G and ABCB1 c.3435C>T in healthy controls from west Algerian population

Gene/SNP	Genotype	n = 472 (%)	Allele	n = 472 (%)
CYP3A5 c.6986A>G	GG	295 (62.5)	G	739 (78.3)
	GA	149 (31.6)	A	205 (21.7)
	AA	28 (5.9)		

**Gene/SNP**	**Genotype**	**n = 472 (%)**	**Allele**	**n = 472 (%)**

ABCB1 c.3435C>T	CC	206 (43.6)	C	619 (65.6)
	CT	207 (43.9)	T	325 (34.4)
	TT	59 (12.5)		

n, number; %, frequency

The ABCB1 c.3435C>T genotypes showed 43.6% as frequency for the homozygote 3435CC, 12.5% for the homozygote 3435TT and 43.9% for the heterozygote 3435CT. The allelic frequencies for 3435C and 3435T were respectively 65.6% and 34.4%. The genotype distributions were quietly similar to the expected distributions in Hardy-Weinberg equilibrium (X2=2.41, P=0.30 for c.6986A>G and X2=0.38, P=0.82 for c.3435C>T) (table2).

### Genetic variants in the Algerian population compared to other populations

Table 3 compares the minor allele frequencies (MAF) of the ABCB1 c.3435C>T polymorphism between the Algerian population and other populations available in the 1000 Genomes Project. The table also presents the allelic and genotypic distribution of selected variants, along with corresponding p-values, among Algerians and other populations.

**Table 3:** Allelic distribution of the c.3435C>T polymorphism of the ABCB1 gene in the population studied and in other populations around the world

Population	Algerian	African	European	American	South Asian	East Asian	All
(n)	(472)	(661)	(503)	(347)	(489)	(504)	(2504)
**CC**	0.436	0.723	0.231	0.329	0.192	0.377	0.396

**CT**	0.439	0.254	0.503	0.487	0.466	0.450	0.417

**TT**	0.125	0.023	0.266	0.184	0.342	0.173	0.187

**p-value**		<0.001	0.003	0.267	<0.001	0.613	0.506

**C**	0.656	0.850	0.482	0.572	0.425	0.602	0.600

**T**	0.344	0.150	0.518	0.428	0.575	0.398	0.400

**p-value**		0.002	0.012	0.21	0.001	0.40	0.40

The distribution of allelic and genotypic frequencies of the CYP3A5 c.6986A>G polymorphism in the Algerian population is shown in Table 4. This table also includes comparisons with other populations worldwide, providing insights into interethnic variations of this polymorphism.

**Table 4:** Allelic distribution of the c.6986A>G polymorphism of the CYP3A5 gene in the population studied and in other populations around the world

	Algerian	African	European	American	South Asian	East Asian	All
(n)	(472)	(661)	(503)	(347)	(489)	(504)	(2504)
**GG**	0.625	0.036	0.891	0.651	0.456	0.506	0.470

**GA**	0.316	0.287	0.105	0.291	0.423	0.415	0.304

**AA**	0.059	0.676	0.004	0.058	0.121	0.079	0.227

**p-value**		<0.001	<0.001	0.91	0,050	0,235	<0.001

**G**	0.783	0.180	0.943	0.797	0.668	0.713	0.621

**A**	0.217	0.820	0.057	0.203	0.332	0.287	0.379

**p-value**		<0.001	0.001	0.728	0.082	0.256	0.014

## Discussion

We investigated the genetic distribution of CYP3A5 c.6986A>G and ABCB1 c.3435C>T polymorphisms in the western Algerian population, and compared the genotypes and allele distributions with diverse ethnic groups. The allele frequency distribution of the ABCB1 c.3435 C>T polymorphism was compared to African, European, Asian and American.

No significant difference was observed between our frequencies for 3435T (0.34) and those of the American (0.42) and East Asian populations (0.39) (p>0.025). On the contrary, the frequencies observed in the Caucasian (0.51), African (0.15) and South Asian populations (0.57) do not agree with our findings p<0.025).

However, the allelic frequency in the Moroccan population (T=0.34) showed consistent results with those found in our study (p>0.025). 13

For the CYP3A5 c.6986A>G, the frequency of the 6986A in our sample is 0.21, which is close to that perceived in the American (0.20), East and South Asian populations (0.28, 0.33 respectively), but differ significantly from the African (0.82) and Caucasian (0.05) (p<0.05).

Compared to the North African populations, our results are consistent with those of the Moroccan and Tunisian populations, where the frequency of the 6986A was 0.26 and 0.12 respectively (p>0.05). 14,15

For the ABCB1 c.3435C>T polymorphism, many studies have demonstrated that the 3435TT genotype is associated with decreased doses of tacrolimus in people with Kidney Transplantation as compared to 3435CT and 3435CC genotypes in the European and Asian populations 16,17. For toxicity, a meta-analysis including cancer patients receiving docetaxel have found a significant association between ABCB1 3435TT genotype and a higher risk of developing hematological and neurotoxicity 18. In the Algerian population, The ABCB1 3435T polymorphism was not associated with adverse drug reactions of methotrexate therapeutic outcome in rheumatoid arthritis patients 19.

In results, 3435T allele is correlated with high toxicity 20. Contrary to European, African populations with low allele frequencies have a lower risk of developing toxicity 21,22. However, for the efficacy, the standard prescribed dose will be adequate for the Caucasian populations but too low for African populations and therefore ineffective.

The CYP3A5 is also implicated in the metabolism of a large number of different drugs, some studies have observed that the 6986AA and 6986AG genotypes are associated with increased dose of tacrolimus and in people with Kidney Transplantation as compared to 6986GG 22,23. Moreover, for the same patients treated with tacrolimus, the 6986GG was associated with increased severity of interstitial fibrosis 24 and nephrotoxicity 25. On the other hand, a study including kidney transplant from Algeria didn't find an association between CYP3A5 c.6986A>G and tacrolimus pharmacokinetics 26. For breast cancer patients, the 6986A allele was associated with increased clearance of docetaxel compared to allele 6986G 27, similarly, genotype 6986GG was found to be associated with decreased response to docetaxel and paclitaxel in people with breast neoplasms as compared to 6986AA genotype^27^.

6986A allele increases the metabolic capacities, thus reducing the effectiveness of the treatment^28^. As the 6986G variant corresponds to a weak expression of the CYP3A5 enzyme, and the frequency of this allele is the most important in our general population, we can suppose that our patients receiving this drug will have a better response, but will present a higher risk of toxicity.

The findings of this study highlight the limitation of insufficient clinical data availability. However, the large cohort size yielded robust results reflecting the distribution of these gene polymorphisms in the general population. Nevertheless, conducting more studies to validate and extend the implications of the pharmacogenetic polymorphisms is essential. Additionally, investigating the functional consequences of these genetic variations on drug metabolism and treatment outcomes would provide valuable insights for personalized medicine approaches.

## Conclusion

Our findings indicate the existence of a difference in the allele distribution of ABCB1 and CYP3A5 variants in the Algerian population compared to the other populations. We demonstrate that 3435T ABCB1 and 6986A CYP3A5 represent the minor allele in our population. These results may contribute to enhance the characterization of pharmacogenetics profiles in the Algerian population and predict the efficacy and toxicity of several drugs on Algerian patients presenting different pathologies in which these genes are involved.
